# Combination of JAK inhibitor and immune checkpoint inhibitor in clinical trials: a breakthrough

**DOI:** 10.3389/fimmu.2024.1459777

**Published:** 2024-10-04

**Authors:** Shiqing Dong, Zhongnan Ma

**Affiliations:** ^1^ School of Life Science, Huaiyin Normal University, Huai’an, Jiangsu, China; ^2^ Department of Pharmacology and Molecular Sciences, Johns Hopkins University School of Medicine, Baltimore, MD, United States

**Keywords:** JAK inhibitor, immune checkpoint inhibitor, combination, immunotherapy, clinical trial

## Introduction

Recently, two back-to-back clinical trials research studies published in *Science*. Zak et al. and Mathew et al. reported that combining these therapeutic approaches led to improved clinical responses compared to immune checkpoint inhibition alone in patients with relapsed or refractory Hodgkin lymphoma and metastatic non-small cell lung cancer (NSCLC), respectively (1, 2).

The landscape of cancer treatment, particularly immunotherapy, has evolved dramatically over the past few decades. Immune checkpoint inhibitors (ICIs), which increase the ability of the immune system to attack cancer cells, are a promising revolution (3). However, not all patients respond to ICIs, leading to the search for combination therapies that can enhance their efficacy (4). A promising approach is the combination of Janus kinase (JAK) inhibitors (JAKi) and ICIs. These studies explored the potential of this combination therapy for the treatment of various cancers. Before these two studies, this combination of therapies in cancer treatment was rarely undertaken. This is because the strategy appears to be paradoxical. Why can JAKi (which suppress immune cell activation and proliferation) increase the immune-mediated elimination of cancer cells?

JAKi are small molecules that block the activity of one or more members of the JAK family of enzymes. These enzymes are critical for the signaling of various cytokines and growth factors involved in hematopoiesis (5), immune function (6), and inflammation (7, 8), neuropathology (9). These drugs modulate the immune response by inhibiting JAKs, making them effective for treating autoimmune diseases. JAKi include Ruxolitinib, Itacitinib, and tofacitinib, each with varying specificities for JAK1, JAK2, JAK3, and TYK2 (10).

ICIs, such as nivolumab (anti-PD-1), pembrolizumab (anti-PD-1), and ipilimumab (anti-CTLA-4), block the inhibitory pathways that limit T-cell activation. These pathways include PD-1/PD-L1 and CTLA-4, which are often exploited by tumors to evade the immune system. By blocking these checkpoints, ICIs enhance T-cell responses against cancer cells, leading to improved antitumor activity (11).

In general, JAKi decrease T-cell activity in most autoimmune diseases. However, it is beneficial for patients with high T-cell activation in a tumor environment with conventional cognition. Therefore, only a few groups have attempted to combine immunotherapy with inflammatory inhibitors for cancer therapy. It is a new mechanism that these two *Science* papers show that JAKi can improve antitumor responses, implying that a new rationale will be found.

Here, the authors hypothesized that JAKi can be combined with ICIs. This combination is based on complementary mechanisms. While ICIs boost the immune system’s ability to recognize and attack cancer cells, JAKi modulate the immune environment to reduce factors that limit this response rather than directly targeting immune cells. 1). Enhancing T-Cell Activity: JAKi can help overcome the immunosuppressive environment within tumors by reducing the activity of myeloid-derived suppressor cells (MDSCs) and regulatory T-cells (Tregs). This enhances the activation and proliferation of cytotoxic T-cells, which are crucial for effective antitumor responses. 2). Reducing Immune Resistance: Chronic inflammation and persistent interferon signaling within the tumor microenvironment can lead to T-cell exhaustion. JAKi can reset chronic signaling, reducing T-cell exhaustion and improving the efficacy of ICIs.

Zak et al. explored the use of Ruxolitinib, a JAK1/2 inhibitor, in combination with nivolumab (anti-PD1) in mouse models of chronic lymphocytic choriomeningitis virus (LCMV) infection, a model for chronic viral infections and cancer. The study found that Ruxolitinib administration resulted in increased numbers of antigen-specific CD8^+^ T-cells and reduced the viral load. Importantly, the combination of Ruxolitinib and nivolumab led to significant reductions in tumor growth compared to either treatment alone. A phase 1 trial conducted by Zak et al. assessed the efficacy of Ruxolitinib in combination with nivolumab in 19 patients with relapsed or refractory Hodgkin lymphoma who had previously received ICIs. The trial showed an impressive overall survival rate of 87% at two years, compared to 23.8% with ICIs therapy alone. Patients receiving combination therapy exhibited decreased numbers of myeloid progenitors and MDSCs, increased cytokine-producing CD8^+^ T-cells, and improved antitumor immune responses.

Similarly, Mathew et al. investigated the combination of Itacitinib, a JAK1 inhibitor, and pembrolizumab (anti-PD1) in a mouse model of NSCLC. Combination therapy significantly improved survival and reduced tumor burden, with treated mice showing increased T-cell infiltration and reduced expression of exhaustion markers. They conducted a phase 2 clinical trial combining Itacitinib with pembrolizumab in 21 treatment-naïve patients with NSCLC. The trial demonstrated a median progression-free survival of nearly two years, significantly longer than the 6.5 to 10.3 months observed with pembrolizumab alone in other trials. Combination therapy led to a proliferative burst of CD8^+^ T-cells and reduced T-cell exhaustion.

The mechanisms underlying the enhanced efficacy of the combination of JAK inhibitors with ICIs are multifaceted and involve several key processes: 1). Reversing T-Cell Exhaustion; 2). Modulating the Tumor Microenvironment; and 3). Enhancing Antigen Presentation.

However, combination therapy remains a challenge. 1). Safety and Toxicity, as combining two potent immune-modulating therapies raises concerns regarding increased toxicity and adverse effects. Careful monitoring and management of adverse effects are essential to ensure patient safety. 2). Patient Selection, that is, identifying patients who are most likely to benefit from combination therapy is critical. Biomarkers that predict response to JAKi and ICIs can help treat individual patients. 3). Optimal Dosing and Timing, as determining the optimal dosing and timing of JAK inhibitors in combination with ICIs is crucial for achieving the best clinical outcomes. 4). Resistance Mechanisms, as understanding the mechanisms of resistance to combination therapy is essential for developing strategies to overcome this resistance. Studies exploring the molecular pathways involved in drug resistance may facilitate the development of next-generation therapies.

In summary, the combination of JAKi and ICIs is a promising strategy for enhancing antitumor immunity and overcoming resistance to immunotherapy. Preclinical and clinical studies have demonstrated the potential of this approach in treating relapsed or refractory Hodgkin lymphoma and NSCLC, showing improved survival and tumor control compared to ICI therapy alone (1, 2). The mechanistic insights gained from these studies highlight the importance of reversing T-cell exhaustion, modulating the tumor microenvironment, and enhancing antigen presentation to achieve effective antitumor responses. However, challenges related to safety, patient selection, dosing, and resistance mechanisms must be addressed to fully realize the potential of this combination therapy. It is a very exciting insight and positive result to support combining anti-inflammatory inhibitors and immunotherapy, not restricted to the JAKi with anti-PD1/PD-L1 combination.

## Discussion and prospective

Although these clinical trials provide promising preliminary data, it must be acknowledged that these studies have limitations in terms of sample size and study design. For example, the study by Zak et al. included only 19 patients, which may limit the generality of the results. Moreover, the lack of long-term follow-up data precludes the assessment of the durability of the therapeutic effects and potential long-term side effects, which should be further investigated in subsequent studies.

When discussing the safety of these clinical trials, it is crucial to analyze the immune-related adverse events associated with combination therapy. For instance, patients may experience severe immune-mediated side effects during treatment, such as aggressive autoimmune diseases and some else emerged inflammatory responses. Several serious immune-related adverse events were reported in the study by Zak et al., which warrants special attention in patient management to ensure safety.

While JAK inhibitors are typically considered to suppress immune cell activation, in specific tumor microenvironments, these drugs can selectively reduce the activity of suppressive immune cells such as myeloid-derived suppressor cells (MDSCs) and regulatory T cells (Tregs), thereby enhancing the activity of cytotoxic T cells. Thus, the role of JAK inhibitors is not merely to suppress immune responses, but to finely tune the tumor microenvironment to promote antitumor immunity. It may be a new strategy for combination (anti-inflammation drugs and anti-tumor growth drugs), such as TNF inhibitor, ACE (Angiotensin-converting enzyme) inhibitor, COX-2 inhibitor.

Biomarkers play a critical role in guiding patient selection and predicting the response to combination therapy with JAKi and ICIs. Current research is exploring various potential biomarkers, such as cytokine profiles within the tumor microenvironment, PD-L1 expression levels (12), and the extent of T-cell infiltration (13), all of which may be used to predict patient response to combination therapy. Future studies need to further elucidate the predictive value of these biomarkers to achieve more personalized cancer treatment.

Although these early clinical trials provide encouraging results, future research should focus on applying this combination therapy to a large range of cancer types. Additionally, exploring the combination of this approach with other emerging therapies, such as cancer vaccines and cellular therapies, could further enhance therapeutic outcomes. As we gain a deeper understanding of the mechanisms underlying the JAKi and ICI combination therapy, the development of next-generation therapies that are more targeted and have lower toxicity will also become an important direction for future research.
